# Drinking Heated Water Improves Performance via Increasing Nutrient Digestibility and Ruminal Fermentation Function in Yak Calves

**DOI:** 10.3390/ani13132073

**Published:** 2023-06-23

**Authors:** Tianxu Liu, Chenxi Gao, Shenfei Long, Qianqian Wang, Tengfei He, Zhenlong Wu, Zhaohui Chen

**Affiliations:** State Key Laboratory of Animal Nutrition, College of Animal Science and Technology, China Agricultural University, Beijing 100193, China; liutianx@cau.edu.cn (T.L.); 17606944398@163.com (C.G.); longshenfei@cau.edu.cn (S.L.); wqq@cau.edu.cn (Q.W.); hetengfei@cau.edu.cn (T.H.); wuzhenlong@cau.edu.cn (Z.W.)

**Keywords:** yak calves, heated water, performance, nutrient digestibility, ruminal function, serum index

## Abstract

**Simple Summary:**

This study aims to explore whether drinking heated water is able to increase the performance of yak calves. We collected and analyzed data from a two-month experiment on yak calves. The results show that compared with drinking cold water, drinking heated water significantly improved the apparent total tract digestibility (ATTD) and ruminal fermentation function. In addition, we found that heated water significantly improved the immune function of the body through serum indexes, and the nutrients in the blood were significantly increased compared with cold water. Furthermore, the serum hormones were also changed by heated water, with higher levels of hormones related to growth. Overall, drinking heated water in the cold season could increase the performance of yak calves in terms of better ATTD, ruminal function and immunity.

**Abstract:**

This study was conducted to investigate the effects of heated water intake on the growth performance, serum biochemical indexes, apparent total tract digestibility (ATTD) of nutrients and ruminal fermentation function of yak calves in winter. A total of 24 yaks (59.09 ± 3.181 kg) were randomly selected and divided into a cold water (fluctuated with the temperature of test sites at 0–10 °C) group (CW) (58.58 ± 3.592 kg) and a heated water (20 °C) group (HW) (59.61 ± 2.772 kg). After 2 months of the experiment, body weight, serum biochemical indexes, ruminal fermentation characteristics and ATTD were measured. The results showed that drinking heated water increased (*p* < 0.05) the total weight gain and average daily gain of yaks compared with those drinking cold water. Heated water increased (*p* < 0.05) the levels of immune globulin M, interleukin-6, triiodothyronine, tetraiodothyronine and growth hormone compared with cold water. In addition, yaks drinking heated water showed higher (*p* < 0.05) ATTD of crude protein and ether extract, as well as increased (*p* < 0.05) content of total protein, albumin and urea nitrogen in serum than those drinking cold water. Compared with cold water, heated water showed increased (*p* < 0.05) total volatile fatty acids, acetic acid and propionic acid, and a reduced (*p* < 0.05) acetic acid to propionic acid ratio (*p* < 0.05). In conclusion, drinking heated water at 20 °C could improve performance via increasing nutrient digestibility and ruminal fermentation function in yak calves.

## 1. Introduction

Yaks (*Bos grunniens*), belonging to the family of Bovidae within the genus *Bos*, originate from the Qinghai–Tibet Plateau, where the altitude is high (2000–5000 m) and the climate is relatively harsh [1]. Because of geographical limitations, yaks are still mainly raised using natural grazing methods, which causes low efficiency in the yak breeding industry [2]. The pasture begins to wither in October due to the high altitude and the low air temperature, and adult female yak lose 25–30% of their weight during the cold season [3]. Global warming leads to the occurrence of extreme climate conditions. In recent years, more and more researchers have paid attention to the effects of cold winter weather on the growth, health and welfare of ruminants [4]. Therefore, it is necessary to address the unsolved question of losing weight in winter.

A previous study showed that the performance of beef cattle in winter is lower, and the negative effects of the cold increase the death rate and reduce the meat quality and immunity [5,6]. Since calves have a weaker digestive system and lower immunity than adult cattle, during the cold season, they are susceptible to diseases of the digestive tract such as diarrhea [7,8]. Additionally, cold environments can negatively affect animal welfare and body health [9]. There are many approaches for alleviating the hazards of cold conditions for calves, and providing heated drinking water seems to be economical viable [10,11]. One study proved that drinking heated water could enhance the efficiency of production because of the better rumen situation and reduction in the expenditure of energy [12]. Compared with 10 °C and 20 °C, dairy cows drinking water at 35 °C was more beneficial [13]. Another experiment found that water of a constant temperature of 17.69 °C could significantly increase the average daily gain (ADG) by 0.36 kg compared with a control group of beef cattle drinking water at 10.18 °C [14].

The rumen is an important organ that plays a key role in nutrient absorption and energy utilization in ruminants. Previous studies indicate that ambient temperature influences the internal environment of the rumen and digestibility, therefore causing changes in the concentration of volatile fatty acids (VFAs) [15]. Nutrients digested in the rumen are transported into the bloodstream; thus, the serum index can be an indicator of the nutrient utilization of animals [16]. In addition, previous studies indicate that the serum indexes can be used to evaluate the level of injury and change significantly in animals exposed to cold environments [17]. Therefore, exploring the differences in ruminal fermentation characteristics and serum indicators would be helpful for understanding the internal mechanism to some extent.

There are currently few studies about the methods of rearing yak calves and influences of suitable drinking water temperature. Therefore, this study aims to investigate the effects of heated drinking water on the growth performance, serum indexes and ruminal fermentation function of yak calves in cold months.

## 2. Materials and Methods

### 2.1. Ethics Statement

**Figure 1:**
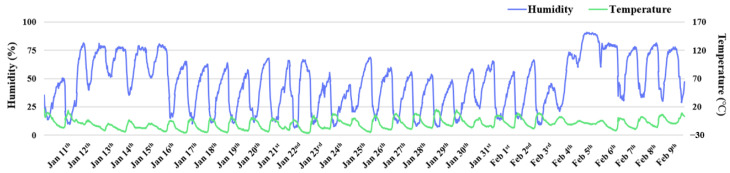
Humidity and temperature curves of test sites. Notes: The blue line represents the humidity of test sites and the green line represents the temperature of test sites.

This study was conducted between January 2023 and May 2023 at the Qinghai Yak Breeding and Extension Service Center of Qinghai Province, China, and was approved by the China Agricultural University Laboratory Animal Welfare and Animal Experimental Ethical Committee (No. AW22503202-1-1). The temperature and humidity of two test sites in the cold season (11 January 2023 to 9 February 2023) were recorded and merged (Figure 1). The temperature in the cold season fluctuated between around −25.5 °C and 18.3 °C, while the humidity fluctuated between 5.5% and 96.9%. As shown in Table 1, the environmental indexes had large fluctuations during the experiment.

### 2.2. Animals and Experimental Design

This experiment was conducted at the Qinghai Yak Breeding and Extension Service Center, Qinghai Province, China (altitude approximately 2800 m; 37°26′ N latitude and 101°36′ E longitude). A total of 24 female yak calves (59.09 ± 3.181 kg) of similar age (6 months old) were assigned randomly to two groups, differing by the temperature of drinking water: (I) 12 yaks (initially weighed 58.58 ± 3.592 kg) were given cold drinking water without any heating (at 0–10 °C) (CW); (II) 12 yaks (initially weighed 59.61 ± 2.772 kg) were given constant-temperature drinking water all day (at 20 °C) (HW). All the animals were dewormed before the experiment. Yaks in the two groups were housed in 2 pens, with 12 yaks in each pen (20 × 8 m). Each pen also had a fenced area (20 × 6 m) used as a field of activity for the yaks during the daytime. In this study, the experimental diets were consistent with the yak breeding farm original diets, and the feed composition and nutrient levels are shown in Table 2. All the animals were fed with total mixed ration (TMR) twice a day at 09:00 and 17:00, and it yaks were free to drink all day.

### 2.3. Sample Collection

Two temperature and humidity recorders (Beijing Qingzhengfenhao Technology Co., Ltd., Beijing, China) were placed in each pen to record the climate indexes in the cold season of the experimental areas in Qinghai province. All the yaks were weighed in the morning before feeding on day 0, day 30 and day 60. The average daily gain (ADG) from day 0 to day 30, day 30 to day 60 and day 0 to day 60 was calculated after the experiment as follows:

$$\mathrm{ADG}\;\left(\mathrm{from}\;\mathrm{day}\;A\;\mathrm{to}\;\mathrm{day}\;B\right)=\frac{{\mathrm{Weight}}_{\mathrm{day}\;B}-{\;\mathrm{Weight}}_{\mathrm{day}\;A}}{\mathrm{Days}\;\mathrm{of}\;\mathrm{experiment}}$$

Notes: A and B represent the beginning and end, respectively.

Before morning feeding, blood samples were collected from the jugular vein of each yak (*n* = 12) on day 61 and immediately centrifuged at 3000 rpm for 15 min at 4 °C in a 10 mL vacuum collection vessel (Kangjian Medical Apparatus Co., Ltd., Taizhou, China) to obtain serum, which was stored at −20 °C until testing. Twelve rumen fluid samples (*n* = 6) were collected 2 h after morning feeding via esophageal intubation on day 62. First, 30 mL was discarded to minimize contamination from the saliva, the 20 mL was collected and the pH of the fluid was determined at once. These rumen fluid samples were filtered with four layers of sterile cheesecloth and then divided into three 2 mL sterile cryogenic vials (NEST Biotechnology Co., Ltd., Wuxi, China). The sterile cryogenic vials were stored at −80 °C for NH_3_-N and VFA analysis. On the same day, the feed of the animals was collected for feed composition analysis and feces was collected in bags for digestibility analysis.

### 2.4. Digestibility Analysis

The evenly mixed samples were dried at 65 °C to determine the initial water content and crushed through a 40-mesh sieve to determine nutrition. The contents of dry matter (DM), crude protein (CP), ether extract (EE) and crude ash (CA) in diets and feces were determined according to the method of the Association of Official Analytical Chemists (AOAC) [18]. DM was determined by drying at 105 °C for 24 h. The content of N was measured using the Kjeldahl method, and CP was calculated as  N × 6.25. EE was determined using the weight loss of DM after 8 h of extraction with ether in Soxhlet extraction apparatus. Additionally, through burning in a muffle furnace at 580 °C, the content of CA was measured. The contents of neutral detergent fiber (NDF), acid detergent fibers (ADF) and acid insoluble ash (AIA) in the feed and feces were determined according to the method of Van Soest et al. [19]. Apparent nutrient digestibility was determined using the AIA method, and the apparent digestibility of a given nutrient was calculated using the following formula [20]:

$$\mathrm{Apparent}\;\mathrm{digestibility}\;\left(\%\right)=100\times \left[1-\frac{A1\times \;N2}{A2\times \;N1}\right]$$

Notes: A1 and A2 represent the contents of AIA of feed and feces, respectively. N1 and N2 represent the contents of nutrients of feed and feces, respectively.

### 2.5. Fermentation Analysis

HPLC-grade N-butanol was taken and placed in a centrifuge tube, and an appropriate amount of acetic acid, propionic acid, butyric acid, isobutyric acid, valeric acid, isovaleric acid and hexanoic acid were added in turn to obtain a standard solution after mixing. VFAs were extracted and analyzed though gas chromatography–mass spectrometry (GC-MS) using an Agilent 8890B GC (Agilent, NY, USA) with an Agilent 5977B/7000D mass selective detector. HP-FFAP (30 m × 0.25 mm × 0.25 μm) capillary columns were used for separation at a constant flow rate (1 mL/min). The gas column temperature was 80 °C, then it increased to 120 °C at a rate of 40 °C/min, later increased to 200 °C at a rate of 5 °C/min and was finally held at 220 °C for 3 min. Masshunter software (v10.0.707.0, Agilent, NY, USA) was used to identify and quantify the compounds, and the concentration results were calculated.

### 2.6. Serum Index

Immune globulin A (IgA), immune globulin G (IgG), immune globulin M (IgM), interleukin-1β (IL-1β), interleukin-6 (IL-6) and tumor necrosis factor (TNF-α) in blood serum were determined using Elisa kits (Beijing Laibotairui Technology Co., Ltd., Beijing, China) according to the manufacturer’s instructions. Concentrations of serum triiodothyronine (T3), tetraiodothyronine (T4), cortisol (COR) and growth hormone (GH) were measured using the radioactive immunity analysis method (North Biotechnology Research Co., Ltd., Beijing, China). Glucose (GLU), total cholesterol (TC), triglycerides (TG), TP, albumin (ALB) and blood urea nitrogen (BUN) were detected using assay kits (Zecheng Biotechnology Co., Ltd., Taizhou China) according to the instructions.

### 2.7. Statistical Analysis

All data of this experiment were statistically analyzed using the unpaired *t*-test with GraphPad Prism (GraphPad prism 9.5.0., San Diego, CA, USA), and the results are presented as mean ± standard error. A *p* ≤ 0.05 was considered statistically significant and a tendency was declared at 0.05 ≤ *p* ≤ 0.10.

## 3. Results

### 3.1. Growth Performance

**Figure 2:**
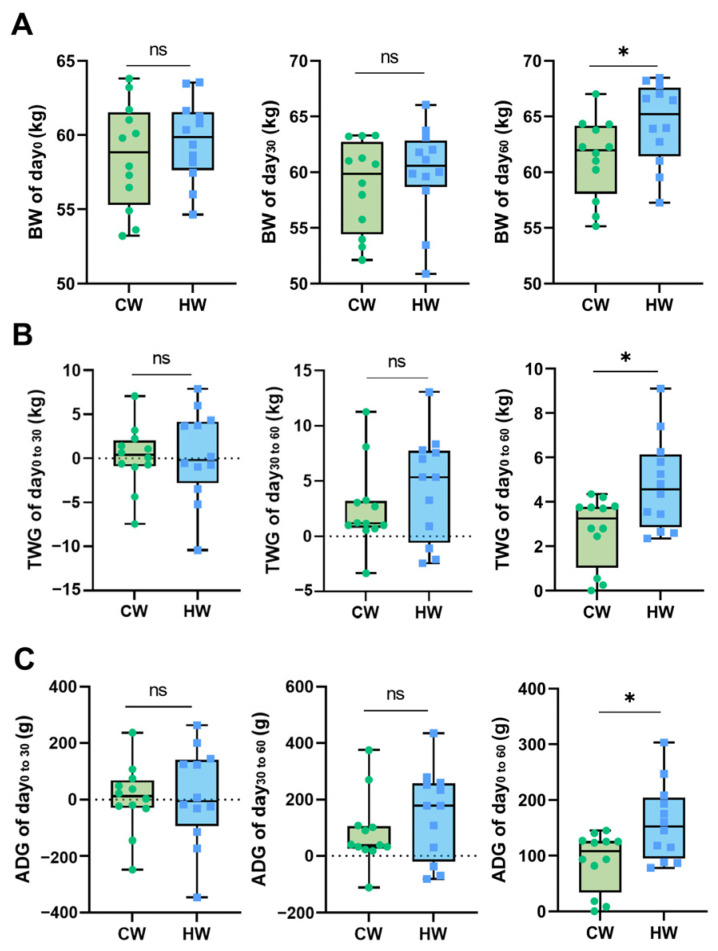
Effects of heated water on growth performance in yak calves (*n* = 12). Notes: (**A**) Body weight (BW/kg) of different stages of experiment. (**B**) Total weight gain (TWG/kg) of different stages of experiment. (**C**) Average daily gain (ADG/g) of different stages of experiment. “*” means the difference between two groups is significant (*p* < 0.05) and “ns” means the difference between two groups is not significant (*p* > 0.05).

The growth performance of yak calves is shown in Figure 2. When the experiment began, the difference in the initial weight of yaks was not significant between the two groups (58.58 ± 3.592 kg for CW and 59.61 ± 2.772 kg for HW; *p =* 0.440), which demonstrates the correctness of grouping. At the end of this feeding period, heated water had significantly influenced the final body weight (BW) of yaks (61.28 ± 3.591 kg for CW and 64.40 ± 3.665 kg for HW; *p =* 0.047). Animals drinking heated water had better total weight gain (TWG) after the experiment, and the increase was extremely significant (2.700 ± 1.578 kg for CW and 4.795 ± 2.080 kg for HW; *p =* 0.011). Moreover, the average daily gain (ADG) (day 0 to day 60) of the HW group significantly increased compared with the CW group (90.00 ± 52.59 g for CW and 159.9 ± 69.35 g for HW; *p =* 0.003).

### 3.2. Apparent Total Tract Digestibility (ATTD) of Nutrients

It can be observed in Table 3 that there were extremely significant differences in the contents of CP, EE and CA (*p* < 0.01). However, the different treatments had no significant effect on the contents of NDF and ADF.

### 3.3. Ruminal Fermentation Characteristics

As shown in Table 4, there were no significant differences between the two treatments in terms of pH, butyric acid, isovaleric acid or hexanoic acid. However, an increasing tendency of the concentration of isovaleric acid in rumen fluid could be found for the animals drinking heated water (*p =* 0.096). The concentration of NH_3_-N, total VFAs, acetic acid and isobutyric acid of the HW group were significantly higher than those of the CW group (*p* < 0.05), and propionic acid, valeric acid and A/P (acetate/propionate) in particular show differences between the two groups (*p* < 0.01).

### 3.4. Serum Immune Indicator

**Figure 3:**
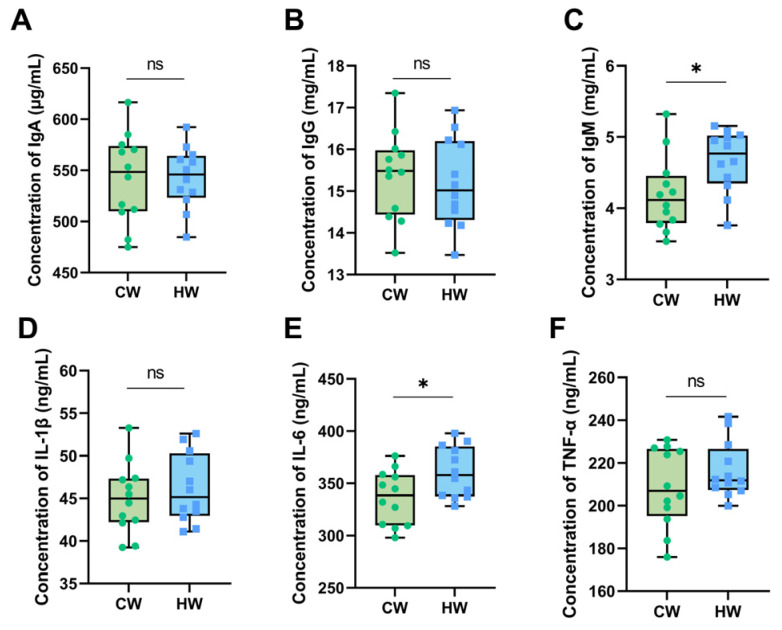
Effects of heated water on serum immune indicators in yak calves (*n* = 12). Notes: (**A**) Concentration of IgA (μg/mL). (**B**) Concentration of IgG (mg/mL). (**C**) Concentration of IgM (mg/mL). (**D**) Concentration of IL-1β (ng/mL). (**E**) Concentration of IL-6 (ng/mL). (**F**) Concentration of TNF-α (ng/mL). “*” means the difference between two groups is significant (*p* < 0.05) and “ns” means the difference between two groups is not significant (*p* > 0.05).

The serum immune indicators of yak calves are summarized in Figure 3. There were no significant differences in the concentration of IgA, IgG, IL-1β or TNF-α (Figure 3A,B,D,F). However, the concentrations of IgM and IL-6 (Figure 3C,E) show significant differences between the two groups (*p* < 0.05).

### 3.5. Serum Routine Indicator

**Figure 4:**
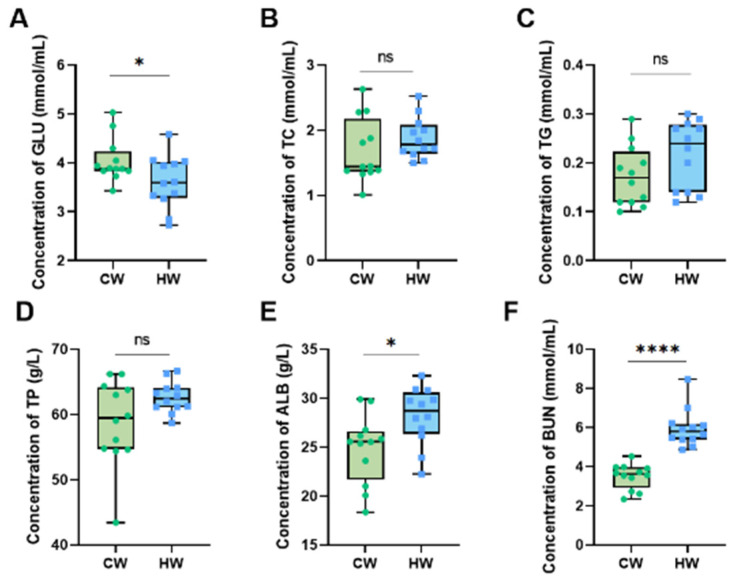
Effects of heated water on serum routine indicators in yak calves (*n* = 12). Notes: (**A**) Concentration of GLU (mmol/mL). (**B**) Concentration of TC (mmol/mL). (**C**) Concentration of TG (mmol/mL). (**D**) Concentration of TP (g/L). (**E**) Concentration of ALB (g/L). (**F**) Concentration of BUN (mmol/mL). “****” means the difference between two groups is extremely significant (*p* < 0.0001), “*” means the difference between two groups is significant (*p* < 0.05) and “ns” means the difference between two groups is not significant (*p* > 0.05).

As demonstrated in Figure 4, the serum routine indicators TC, TG and TP (Figure 4B,C,D) show no significant differences, while the concentrations of GLU and ALB (Figure 4A,E) in the constant heated water group are significantly higher than in the cold water group (*p* < 0.05). Moreover, drinking heated water had an extremely significant effect on the concentration of BUN (Figure 4F) compared with the CW group (*p* < 0.01).

### 3.6. Serum Hormone

**Figure 5:**
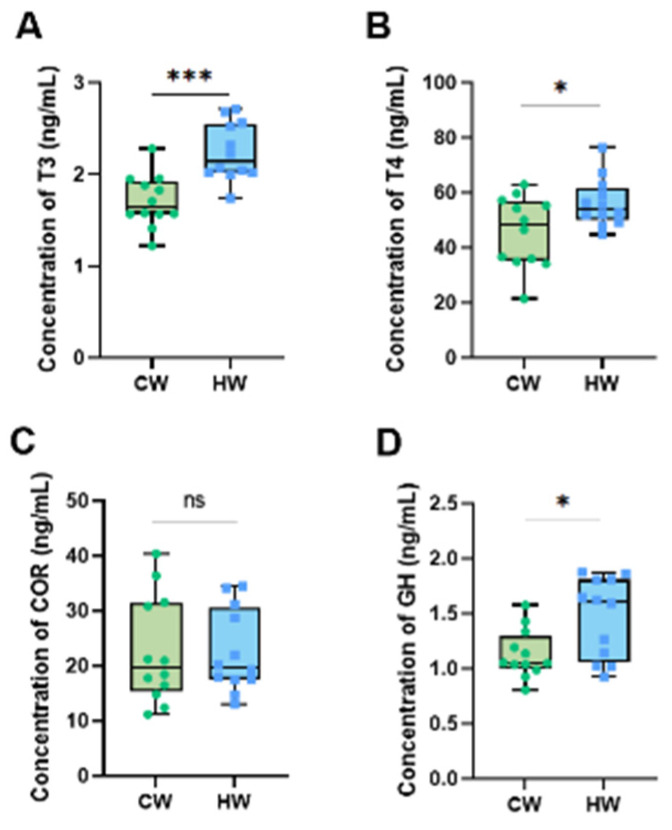
Effects of heated water on serum hormones in yak calves (*n* = 12). Notes: (**A**) Concentration of T3 (ng/mL). (**B**) Concentration of T4 (ng/mL). (**C**) Concentration of COR (ng/mL). (**D**) Concentration of GH (ng/mL). “***” means the difference between two groups is extremely significant (*p* < 0.001), “*” means the difference between two groups is significant (*p* < 0.05) and “ns” means the difference between two groups is not significant (*p* > 0.05).

According to the results (Figure 5), it is clear that the contents of T4 and GH (Figure 5B,D) were significantly raised in the HW group compared with the CW group (*p* < 0.05). Furthermore, the concentration of T3 (Figure 5A) was extremely significantly increased by drinking heated water (*p* < 0.01).

## 4. Discussion

It is well known that growth performance results from the combined effects of nutrient levels and environmental controls during animal rearing [20,21]. In this study, the treatment of drinking heated water significantly increased the TWG and ADG. At the end of the experiment, the BW and ADG of yak calves in the HW group were 7.90% and 76.69% higher than those in the CW group, respectively. Previous studies showed that drinking water at 14–18 °C in winter significantly increased the water intake of beef cattle, and the water temperature of 16–18 °C is most conducive to improving the performance of beef cattle; moreover, the ADG of beef cattle drinking water at 20 °C was significantly higher than of those drinking water at 4 °C [11,14]. The results of this experiment are consistent with these previous findings, which means that drinking heated water is indeed also useful for improving performance in yak calf rearing.

In the current study, the ATTD of CP, EE and CA showed extremely significant differences, while the ATTD of ADF and NDF were similar. According to previous studies, heat stress could influence the relative abundances of rumen microbes, such as *Fibrolytic Ruminococcaceae*, thus decreasing metabolism and fermentation [22,23]. Therefore, the reason for the difference in digestibility may be that the beneficial composition of rumen microbes improved in the treatment with heated water. The CP and EE of diets are mainly decomposed by microorganisms, and the secondary metabolites produced in the rumen are used by the body to promote growth [24,25]. These results are inconsistent with the increased values of serum protein and VFAs in the HW group. A few reports have found that ruminal pH is essential to the digestibility of ADF and NDF [26,27]. Therefore, the rumen fluid pH, which was not significantly different in this experiment, might be the reason for the lack of difference in the digestibility of ADF and NDF.

Ruminal pH, as an important characteristic in the level of ruminal function, was usually in the normal range of 5.0 to 7.5 [28]. In this study, it was observed that heated water had no significant effect on ruminal pH, which was within the normal limit at 7.023–7.090. A previous study about the effects of different water temperatures on sheep was in agreement with this result, demonstrating that typically, water temperature was not sufficient to alter ruminal pH [29]. The content of NH_3_-N in rumen fluid can reflect the utilization efficiency of protein and non-protein nitrogen in the feed of rumen microorganisms to a certain extent [30]. Therefore, the greater amount of NH_3_-N caused the better synthesis of rumen microbial protein (MCP) and better performance of the HW group compared with the CW group [31]. It is widely known that fiber in forage is degraded by rumen microbes and converted into VFAs, which can provide 70–80% of the energy requirements of the body, and acetic acid, propionic acid and butyric acid were the main components of VFAs in rumen fluid [32,33]. Furthermore, studies have proved that propionic acid can produce GLU through gluconeogenesis, while a reduction in A/P can improve the performance of ruminants [34,35]. In this study, acetic acid, propionic acid, isobutyric acid and valeric acid were significantly increased by drinking heated water, and the ratio of acetic acid to propionic acid (A/P) was extremely significantly decreased, which is consistent with the results of the better performance of yak calves in the CW group.

Blood indicators are widely used to evaluate the nutrient absorption, metabolism and health of animals [36,37]. IgA, IgG and IgM, which could reflect the condition of immunity, are produced by B-cells and can help in recognizing antigens and killing bacteria [38,39]. Thus, a higher amount of immune globulin, within certain limits, would eliminate antigens better in animals’ bodies, which can be beneficial to the maintenance of the internal environment [40]. Previous studies about broilers and mice have found that IL-1, IL-2 and IL-6, often considered inflammatory cytokines, can increase the body’s immunity and improve the level of disease resistance [41,42]. In this study, the levels of IgM and IL-6 in the HW group were greater than in the CW group, which means better body immunity and condition compared with the yak calves drinking cold water.

Significant differences in TP and ALB, the most important nutrition indicators synthesized by the liver, were found in blood serum [43]. According to Salem [44], TP and ALB were low in diseased calves, which means that the yak calves of the HW group in the current study have a better condition in terms of nutrient levels. Another study demonstrated that BUN levels were higher in cattle fed with silage inoculated with complex lactic acid bacteria compared with an untreated group, and this was because of better function and activity of the liver [45]. Furthermore, the increased BUN in serum was associated with better protein absorption, which was also reported in a previous study [22], indicating that the increased BUN in yak serum in the HW group demonstrates better feed CP utilization.

Hormones play a key role in the growth and development of animals [46]. Thyroid hormones, to some extent, could promote growth and development through energy metabolism, influenced by the release and action of GH [47,48,49]. Therefore, the result of the better performance of the HW group showed the consistency of the trend of growth hormones and thyroid hormones, and that the heated water does affect growth performance through kinds of hormones. It is obvious that a body with better physiological conditions can effectively cope with a harsh external environment, which also explains why drinking warm water instead of cold water could increase the ADG of yaks in this experiment [50].

## 5. Conclusions

Drinking constantly heated water at 20 °C in winter could improve the ATTD of feed nutrients, ruminal fermentation function and the immunity of the body, which led to improved performance of yak calves and animal welfare in the cold season. However, the continuous heating of drinking water consumes more energy and increases the economic burden. Therefore, follow-up research can focus on how to increase economic benefits and save electrical energy.

## Figures and Tables

**Table 1:** Environmental indexes of test sites.

Item	Treatment	
	CW	HW
Temperature, °C		
Maximum temperature	16.9	18.3
Average temperature	−9.4	−9.4
Minimum temperature	−25.5	−26.4
Relative humidity, %		
Maximum humidity	87.4	96.9
Average humidity	46.1	45.2
Minimum humidity	6.8	5.5

**Table 2:** Ingredients and nutrients of the experimental diets.

Item	Amount
Ingredients composition, %	
Dried wheat grass	33.6
Dried oat grass	32.0
Corn silage	14.4
Corn	6.0
Wheat	4.0
Wheat bran	2.5
Rapeseed meal	4.0
Soybean meal	1.5
NaCl	0.5
Premix ^1^	1.5
Chemical composition, % of dry matter (DM)	
DM	87.64
Crude protein (CP)	7.31
Ether extract (EE)	1.34
Neutral detergent fiber (NDF)	58.68
Acid detergent fiber (ADF)	33.68
Coarse ash (CA)	9.45

Notes: ^1^ The premix provides following per kilogram: Ca, 6 g; P, 4.5 g; Na, 4.8 g; K, 9 g; Mg, 3.6 g; Cu, 22 mg; Fe, 126.3 mg; Zn, 46.8 mg; Mn, 54.18 mg; VA, 60,000 IU; VD_3_, 25,000 IU; VE, 100 IU. Nutrient levels were measured values.

**Table 3:** Effects of heated water on ATTD of nutrients in yak calves (*n* = 6).

Item	Treatment		***p*-Value**
	CW	HW	
CP, %	20.44 ± 10.090	40.67 ± 6.484	0.002
EE, %	28.33 ± 8.568	48.66 ± 10.170	0.004
NDF, %	69.08 ± 5.246	71.96 ± 3.311	0.283
ADF, %	71.09 ± 4.495	73.40 ± 3.528	0.345
Ca, %	26.27 ± 4.055	35.12 ± 1.691	0.001

**Table 4:** Effects of heated water on ruminal fermentation characteristics in yak calves (*n* = 6).

Item	Treatment		***p*-Value**
	CW	HW	
pH	7.047 ± 0.1193	6.998 ± 0.1570	0.562
NH_3_-N, mg/kg	68.33 ± 56.36	211.7 ± 96.63	0.011
VFAs, mg/kg			
Total VFAs	4509 ± 597.7	5266 ± 433.6	0.031
Acetic acid (A)	2604 ± 313.7	2976 ± 196.4	0.034
Propionic acid (P)	926.2 ± 136.2	1215 ± 157.1	0.007
Butyric acid	743.3 ± 77.07	818.5 ± 70.45	0.108
Isobutyric acid	47.68 ± 13.89	62.62 ± 6.391	0.038
Valeric acid	67.45 ± 11.55	92.83 ± 15.06	0.008
Isovaleric acid	66.25 ± 20.40	82.03 ± 5.005	0.096
Hexanoic acid	15.57 ± 2.349	19.12 ± 7.255	0.281
A/P	2.886 ± 02496	2.497 ± 0.1220	0.006

## Data Availability

The data presented in this study are available on request from the corresponding author.
